# Cloning and Expression of a Cytosolic HSP90 Gene in *Chlorella vulgaris*

**DOI:** 10.1155/2014/487050

**Published:** 2014-03-13

**Authors:** Zhengyi Liu, Lei Zhang, Yang Pu, Zhaopu Liu, Zhiling Li, Yushan Zhao, Song Qin

**Affiliations:** ^1^Key Lab of Marine Biology in Jiang Su, College of Resources and Environmental Science, Nanjing Agricultural University, Nanjing 210095, China; ^2^Yantai Institute of Coastal Zone Research, Chinese Academy of Sciences, Yantai 264003, China; ^3^Shandong Oriental Ocean Sci-Tech Co., Ltd., Yantai 264003, China

## Abstract

Heat shock protein 90 (HSP90), a highly conserved molecular chaperone, plays essential roles in folding, keeping structural integrity, and regulating the subset of cytosolic proteins. We cloned the cDNA of *Chlorella vulgaris* HSP90 (named CvHSP90) by combining homology cloning with rapid amplification of cDNA ends (RACE). Sequence analysis indicated that CvHSP90 is a cytosolic member of the HSP90 family. Quantitative RT-PCR was applied to determine the expression level of messenger RNA (mRNA) in CvHSP90 under different stress conditions. *C. vulgaris* was kept in different temperatures (5–45°C) for 1 h. The mRNA expression level of CvHSP90 increased with temperature from 5 to 10°C, went further from 35 to 40°C, and reached the maximum at 40°C. On the other hand, for *C. vulgaris* kept at 35°C for different durations, the mRNA expression level of CvHSP90 increased gradually and reached the peak at 7 h and then declined progressively. In addition, the expression level of CvHSP90 at 40 or 45 in salinity (‰) was almost fourfold of that at 25 in salinity (‰) for 2 h. Therefore, CvHSP90 may be a potential biomarker to monitor environment changes.

## 1. Introduction

Heat shock proteins (HSPs) that first described in* Drosophila melanogaster *[1], are evolutionarily conserved protein families which are ubiquitous in all eukaryotic organisms. HSPs are essential for cells under both normal and stressed conditions as they participate in diverse processes ranging from cellular homeostasis and signal transduction to development [2]. In addition, HSPs also play key roles in defense responses against various environmental stresses that could potentially damage the cellular and molecular structures in the cells [3]. Therefore, HSPs are highly expressed in all organisms and are one of the most abundant proteins, contributing to 1-2% of total cellular proteins under normal conditions [4]. According to molecular weight, HSPs are mainly classified into six families including HSP40, HSP60, HSP70, HSP90, HSP100, and the small HSPs [5].

Among HSP families, heat shock protein 90 (HSP90) is highly conserved member. It is confirmed to locate in different cellular compartments including the chloroplasts, mitochondria, cytosol, nucleoplasm, and endoplasmic reticulum [6]. Under normal conditions, HSP90's diverse roles in biological processes include regulating cellular physiology, signal transduction, and protein folding, degradation, and transportation [2, 7–9]. And it also can be regulated by various environmental stresses such as heat, salinity, desiccation, light, heavy metal, and arsenite stresses [10–13].

Influenced by cyclic tide, algae mainly living in intertidal zone are periodically exposed to a variety of potentially stressful factors including heat or cold shock, high light, and desiccation [14]. To understand the resistant mechanism, the expression of HSPs genes have been investigated in some macroalgae such as* Chondrus*,* Porphyra*,* Undaria*,* Laminaria*,* Saccharina*,* Fucus,* and* Ulva* [15–22] and a few microalgae such as* Chlamydomonas* and* Haematococcus *[23–27]. These studies indicate that the algal HSPs centrally function in resisting a wide variety of environmental stressors.

*Chlorella vulgaris *is a unicellular green alga growing in fresh water [28]. It can be used as live feed in fish aquaculture for its high content of proteins and fatty acids. In addition, because of its fast growth and low cost,* C. vulgaris* becomes a promising candidate bioreactor for large-scale production of value-added proteins [29].* C. vulgaris* has important economical and ecological values but often confronts environmental adversities, including high temperature and high salinity. Therefore, it is often used as a eukaryotic model in studies on stress responses [30]. However, the role of HSPs in adverse stress resistance mechanism of* C. vulgaris* is yet to be performed.

To better understand the mechanism of response by* C. vulgaris* to different types of environmental stimulation, we obtained a HSP90 complementary DNA (cDNA) of* C. vulgaris* by combining homology cloning with rapid amplification of cDNA ends (RACE) approaches, analyzed in bioinformatics the structural features, homologous relationship, and phylogenetic position of CvHSP90, and investigated the messenger RNA (mRNA) expression levels of CvHSP90 under different stress conditions, using real-time quantitative RT-PCR (qRT-PCR).

## 2. Materials and Methods

### 2.1. Sample Collection and Treatment

*Chlorella vulgaris* was presented from Institute of Oceanology, Chinese Academy of Sciences, and then the* Chlorella* was grown in Erlenmeyer flasks in F/2 medium that was filter-sterilized through 0.22 *μ*m filters (Millipore, USA). Cultures in liquid medium or on plate were grown at 20°C in an artificial climate incubator. Cool-white fluorescent tubes were used to provide irradiance at 200 *μ*mol photons m^−2^ s^−1^ in a long-daylight scheme (12 : 12 h light : dark).

In addition,* C*.* vulgaris* was kept in 40°C for 1 h for extracting total RNA and cloning full-length cDNA of CvHSP90 gene. In our heat shock temperature treatment,* C*.* vulgaris* was kept in different temperatures (5, 10, 15, 20, 25, 30, 35, 40, and 45°C) for 1 h to investigate the thermal effect on the expression level of CvHSP90 mRNA. In heat shock time treatment,* C. vulgaris* was kept at 35°C for different times (0 h, 1 h, 2 h, 3 h, 4 h, 5 h, 6 h, 7 h, 8 h, 9 h, 10 h, 11 h, and 12 h, resp.) to investigate the effects of heat shock times on expression level of CvHSP90 mRNA. In salt concentration challenge treatments,* C*.* vulgaris* was kept at 20°C in different salt concentrations (0, 5, 10, 15, 20, 25, 30, 35, 40, and 45 in ‰) for 2 h to investigate the effects of salt challenges on expression level of CvHSP90.

### 2.2. RNA Extraction

Total RNA extraction from* C*.* vulgaris* was performed using the TRIzol reagent (Invitrogen). The cDNA first-strand was synthesized based on M-MLV RT usage information (Promega) using RQI DNase (Promega)-treated total RNA as template. cDNA mix was diluted to 1 : 50 and stored at −80°C for subsequent fluorescent real-time PCR.

### 2.3. CvHSP90 cDNA Cloning

To amplify the partial fragment of CvHSP90 gene from* Chlorella vulgaris*, two homologous cloning primers P1 and P2 were designed based on the conserved sequence of known HSP90s (Table 1). PCR was performed in a 25 *μ*L reaction volume containing 2.5 *μ*L 10X PCR buffer, 2 *μ*L dNTP (2.5 mmol L^−1^), 1 *μ*L of each primer (10 *μ*mol L^−1^), 0.2 *μ*L (1 U) of Taq polymerase (Promega), 1 *μ*L of cDNA template, and 17.3 *μ*L of PCR-grade water. The PCR temperature profile was 94°C for 5 min followed by 33 cycles of 94°C for 1 min, 56°C for 1 min, 72°C for 1 min, and a final extension step at 72°C for 10 min. The interesting fragment (745 bp) was excised and purified as per agarose gel DNA fragment recovery kit (TaKaRa), cloned into pMD-18T vector (TaKaRa), and sequenced (Sangon, Shanghai, China).

To clone the full-length cDNA of CvHSP90, four specific primers, sense primers P3 and P4, and reverse primers P5 and P6 (Table 1) were designed based on the partial sequence amplified. Nested PCR strategy was applied to clone the 3′ end of CvHSP90 using sense primer P3, P4, and reverse primer adapter, while sense primer oligo(dG)-adaptor and reverse primer P5, P6 were used to obtain the 5′ end of CvHSP90. PCR amplification was performed using the same reaction system as described before. The PCR products were cloned into the pMD18-T simple vector (TaKaRa) and sequenced bidirectionally with primers M13-47 and RV-M (Table 1). The sequencing results were verified for cluster analysis.

### 2.4. Sequence Analysis of CvHSP90

CvHSP90 cDNA sequence was analyzed in the BLAST algorithm at National Centre for Biotechnology Information (http://www.ncbi.nlm.nih.gov/blast) and the deduced amino acid sequence was analyzed with the Expert Protein Analysis System (http://www.expasy.org/). Characteristic domains or motifs were identified using the PROSITE profile database. Identity, similarity, and gap percentages were calculated using FASTA program. The Clustal W program (http://www.ebi.ac.uk/clustalw/) was used for multiple alignment of CvHSP90. An unrooted phylogenetic tree was constructed according to amino acid sequences of the selected HSP90 by using the neighbor-joining (NJ) algorithm embedded in MEGA 3.1 program (http://www.megasoftware.net/) [31] and the programs of Clustal X 1.83 [32]. The bootstrap trials were replicated 1000 times to derive the confidence value for the phylogeny analysis.

### 2.5. Quantitative Analysis of CvHSP90 mRNA Expression

mRNA expression level of CvHSP90 was investigated by real-time PCR amplification on a fast real-time PCR system (Applied Biosystem 7500). A product of 150 bp from cDNA was amplified by two CvHSP90 gene-specific primers P7 and P8 (Table 1), and the PCR product was sequenced to verify the specificity of RT-PCR. An internal control, a 109 bp fragment, was amplified by two *β*-actin primers. The real-time PCR amplifications were carried out in a total volume of 20 *μ*L mixture containing 10 *μ*L SYBR Premix Ex Taq (TaKaRa, Tokyo, Japan), 2 *μ*L cDNA, 0.4 *μ*L each forward and reverse primer (10 *μ*mol/L), 0.4 *μ*L ROX Reference Dye (50X), and 6.8 *μ*L PCR-grade water. The reactions conditions were 94°C for 5 min, followed by 40 cycles of 5 s at 94°C, and 30 s at 60°C. Five independent biological replicates were carried out. At the end of each PCR reaction, dissociation curve of amplicon was analyzed to confirm if only one PCR product was amplified and detected. After the PCR program, SDS software V2.01 (Applied Biosystems) was used to analyze the data, during which a baseline was set automatically for consistency. The expression level of CvHSP90 was analyzed by the 2^−ΔΔCt^ method [33]. All data were expressed as mean ± SE (*n* = 5) in terms of relative mRNA. Furthermore, the data were analyzed by ANOVA (one-way analysis of variance) followed by an unpaired two-tailed* t*-test. Difference was considered significant at *P* < 0.05.

## 3. Results

### 3.1. cDNA Cloning and Sequencing of CvHSP90 Gene

A CvHSP90 fragment (745 bp) was amplified by homologous cloning primers P1 and P2 and confirmed highly similar to other known HSP90s. Two pairs of CvHSP90-specific primers (P3-P4 and P5-P6) that designed in the above sequence were used to clone the full-length cDNA. RACE and nested PCR were performed to amplify the two fragments corresponding to the 3′ and 5′ end of the CvHSP90 cDNA. The full-length cDNA sequence of CvHSP90 was determined 3678 bp by cluster analysis of the above fragments.

### 3.2. Characterization of CvHSP90

**Figure 1:**
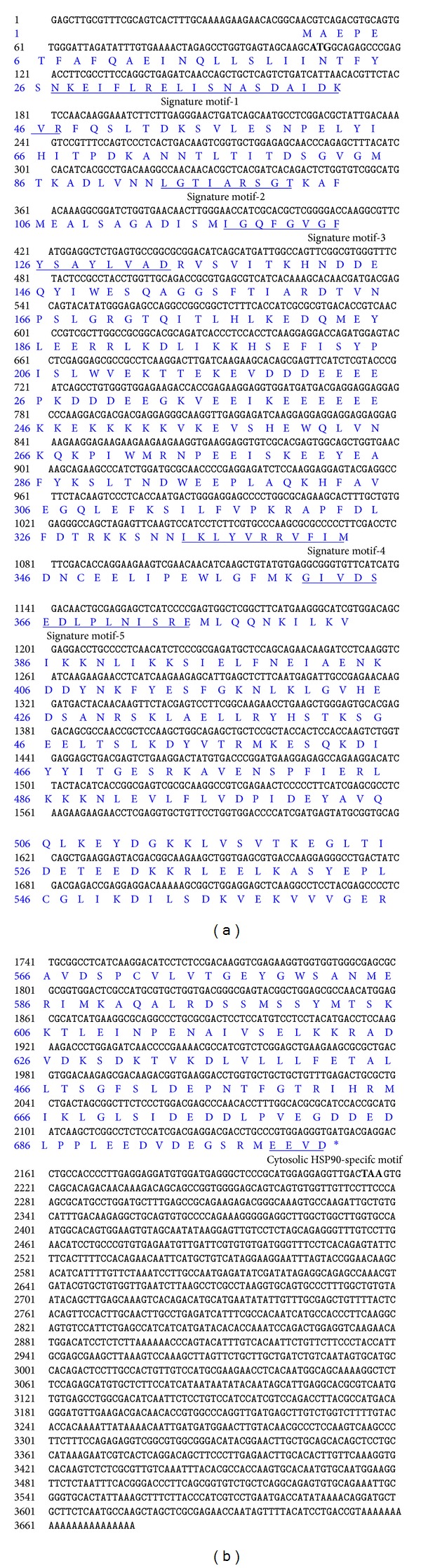
The full length cDNA sequence of CvHSP90 and its deduced amino acid sequence. The nucleotide and deduced amino acid sequence of the open reading frame and flanking region were numbered on the left. The start and stop codons were bold. HSP90 signature motifs and cytosolic HSP90-specific motif were underlined.

The cDNA sequence of CvHSP90 was submitted in GenBank under accession number JQ655149. The full-length cDNA of CvHSP90 was 3678 bp, with a 107 bp 5′ untranslated region (5′UTR), a 1459-bp 3′ untranslated region (3′UTR) with a poly (A) tail, and a 2112 bp open reading frame (ORF) encoding a polypeptide that contained 703 amino acids with estimated molecular mass of 80.71 kDa and an estimated isoelectric point of 4.48. The five typical amino acid blocks of HSP90 protein family, (NKEIFLRE[L/I]ISN[A/S]SDALDKIR, LGTIARSGT, IGQFGVGFYSAYLVA[E/D], IKLYVRRVFI, G[V/I]VDSEDLPLNISRE) and the consensus MEEVD at the C-terminus were highly conserved as indicated in the CvHSP90 sequence (Figure 1) [34]. Meanwhile, SMART program revealed a typical histidine kinase-like ATPases domain in the position 27-182, which is ubiquitous in all HSP90 family members.

### 3.3. Homology Analysis of CvHSP90

**Figure 2:**
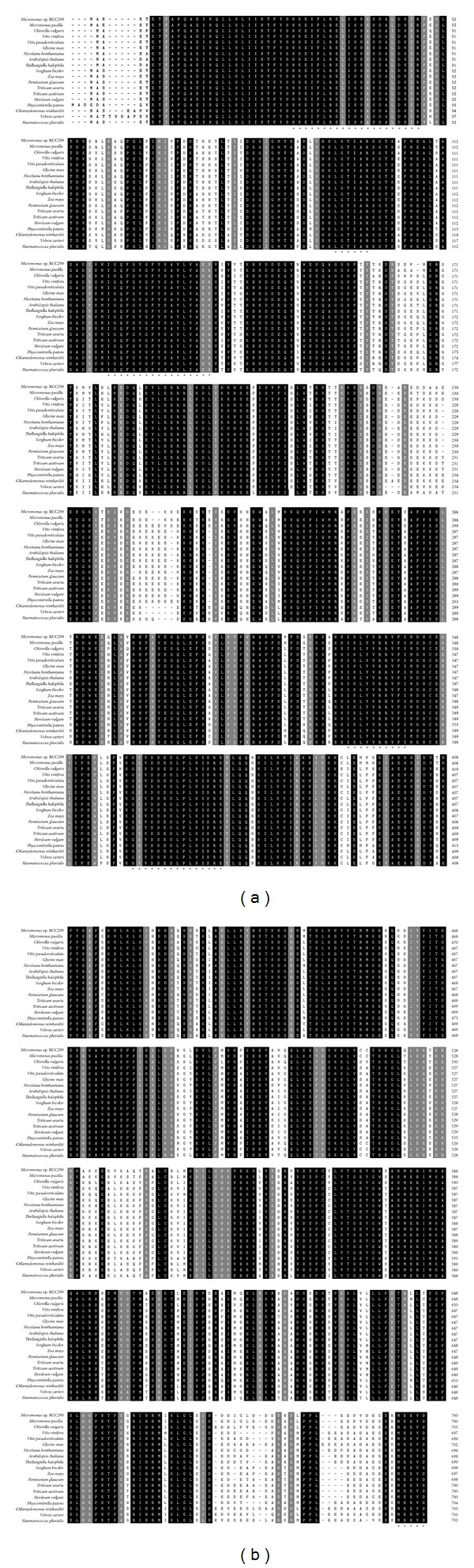
Multiple sequence alignment of the CvHSP90 with other registered counterparts from* Haematococcus pluvialis *(JN627245),* Chlamydomonas reinhardtii* (XP_001695264),* Volvox carteri f. nagariensis* (XP_002947115),* Micromonas *sp.* RCC299* (XP_002499727),* Pennisetum glaucum* (ADP89125),* Hordeum vulgare* (BAJ86355),* Triticum aestivum* (ADF31758),* Triticum urartu* (ADF31773),* Vitis vinifera* (CAN62488),* Thellungiella halophila *(BAJ33984),* Micromonas pusilla CCMP1545* (XP_003058104),* Physcomitrella patens* (XP_001777414),* Sorghum bicolor* (XP_002444804),* Arabidopsis thaliana* (AAN31859),* Vitis pseudoreticulata* (ABW96308),* Nicotiana benthamiana* (AAR12194),* Glycine max* (ADC45395), and* Zea mays* (NP_001170475). The black shaded regions represent identical amino acids among the different species, while the gray shaded regions represent conservative replacements. The HSP90 signature motifs and cytosolic HSP90-specific motif were indicated with asterisks.

Shown by Clustal W program, the deduced amino acid sequence of CvHSP90 shared high homology with those of other known HSP90s (Figure 2). For example, CvHSP90 shared 85% similarity to* Micromonas *sp.* RCC299* HSP90 (XP_002499727) and* Micromonas *sp.* RCC299* HSP90 (XP_002499727), 79% similarity to* Haematococcus pluvialis* HSP90 (JN627245),* Chlamydomonas reinhardtii* HSP90 (XP_001695264), and* Volvox carteri f. nagariensis* HSP90 (XP_002947115), and 78% similarity to* Pennisetum glaucum* HSP90 (ADP89125),* Hordeum vulgare* HSP90 (BAJ86355),* Triticum aestivum* HSP90 (ADF31758),* Triticum urartu* HSP90 (ADF31773),* Vitis vinifera* HSP90 (CAN62488), and* Thellungiella halophila *HSP90 (BAJ33984), and so forth (Table 2). Multiple sequence alignment exhibited high conservation between CvHSP90 and other known HSP90 proteins, especially in the regions of HSP90 family signatures (Figure 2).

**Figure 3:**
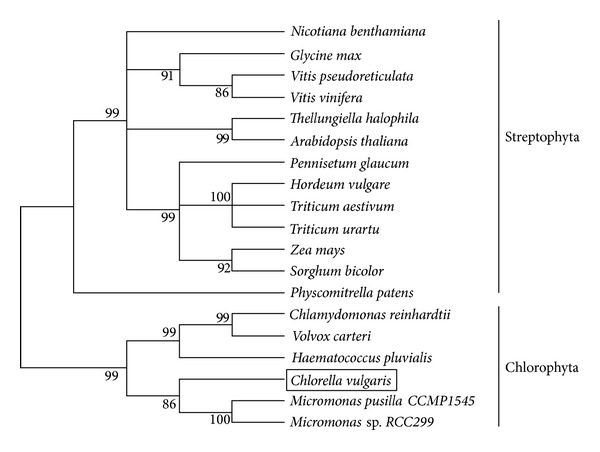
A phylogenetic tree constructed with the neighbor-joining method. The common names and the GenBank accession numbers were the same as those in Figure 2. Numbers at each branch indicate the percentage of times a node was supported in 1000 bootstrap pseudoreplication by neighbor joining.

To evaluate the molecular evolutionary relationships of CvHSP90 against other HSP90s, a phylogenetic tree based on the protein sequences (Figure 3) was constructed by neighbor-joining method. In the phylogenetic tree, the HSP90s are clustered into two major groups in the Streptophyta and Chlorophyta origin. CvHSP90 is clustered first with HSP90 from Mamiellophyceae (*Micromonas *sp.* RCC299* and* Micromonas pusilla CCMP1545*) and then formed a sister group with those of Chlorophyta and further with those of Streptophyta. Relationships displayed in the phylogenic tree agree in overall with traditional taxonomy.

### 3.4. Expression Levels of CvHSP90 under Different Heat Shock Temperatures

**Figure 4:**
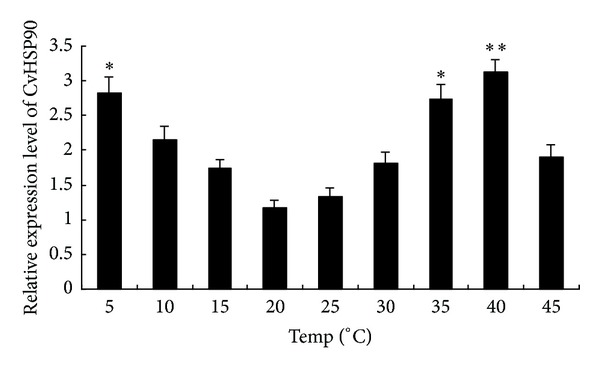
CvHSP90 mRNA expression levels under different heat shock temperatures (5°C, 10°C, 15°C, 20°C, 25°C, 30°C, 35°C, 40°C, and 45°C) for 1 h were analyzed by real-time quantitative reverse transcriptase-polymerase chain reaction. The *β*-actin gene was used as an internal control to calibrate the cDNA template for all the samples. Vertical bars represented the mean ± SE (*N* = 5).

The expression level of CvHSP90 was investigated by real-time quantitative PCR under different stressful conditions. As the optimal growth temperature for* C*.* vulgaris* is 20–25°C, different heat shock temperatures (5–45°C) was scheduled to study the mRNA expression levels of CvHSP90 (Figure 4). In 20°C and 25°C treatment groups, the expression levels of CvHSP90 were relatively low, while in 5°C and 35°C treatment groups were almost 2.5-fold of that in 20°C group (*P* < 0.05). The highest expression level (3-fold of that in 20°C group) was determined at 40°C for 1 h (*P* < 0.01).

### 3.5. Expression Levels of CvHSP90 at Different Heat Shock Times

**Figure 5:**
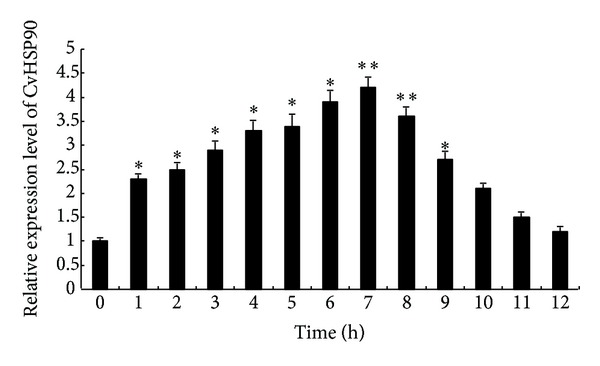
CvHSP90 mRNA expression levels in different heat shock times (0 h, 1 h, 2 h, 3 h, 4 h, 5 h, 6 h, 7 h, 8 h, 9 h, 10 h, 11 h, and 12 h) at 35°C were analyzed by real-time quantitative RT-PCR. CvHSP90 mRNA expression was normalized to the control group, and *β*-actin gene was used as internal control to calibrate the cDNA template for all the samples. Each bar represents the mean value from five determinations with standard error. Significant differences across control were indicated with an asterisk at *P* < 0.05 and two asterisks at *P* < 0.01.

In this study, we found that a long time (over 8 h) exposure at over 40°C would be fatal to* C*.* vulgaris*. Therefore, the organism was kept at 35°C for different hours to find the impact of different heat shock times on the expression level of CvHSP90. As shown in Figure 5, in 35°C, CvHSP90 mRNA expression level increased gradually and reached the maximum (4.2-fold of that of blank group) at 7 h (*P* < 0.01) and then declined progressively to the original level at 12 h. Under a heat shock, the expression level of CvHSP90 was observed significantly different at 1, 2, 3, 4, 5, 8, and 9 h from that of the blank group (*P* < 0.05).

### 3.6. Expression Levels of CvHSP90 under Different Salt Concentrations

**Figure 6:**
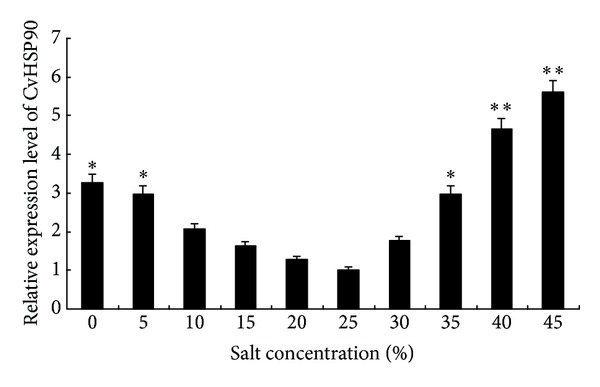
CvHSP90 mRNA expression levels relative to *β*-actin mRNA levels under stress of different salt concentrations analyzed by real-time quantitative RT-PCR. The *β*-actin gene was used as an internal control to calibrate the cDNA template for all the samples. Vertical bars represented the mean ± SE (*N* = 5). Significant differences across control were indicated with an asterisk at *P* < 0.05 and two asterisks at *P* < 0.01.

The expression levels of CvHSP90 at different salt concentrations for 2 h were detected (Figure 6). The expression level of CvHSP90 at 10, 15, 20, and 30 in salinity (‰) for 2 h showed no significant difference from that at 25 in salinity. However, the expression level at salinity over 25 increased significantly (*P* < 0.05). The expression levels at 40 or 45 salinity were almost 4-fold of that at 25 salinity for 2 h (*P* < 0.01). The results show that* C*.* vulgaris *responded strongly to high salinity stress.

## 4. Discussion

HSP90, as an important member of HSPs, centrally functions in various biological processes in the presence and absence of stresses, including biogenesis, folding, transport, degradation, and prevention of misfolding and aggregation of cellular proteins and signal transduction [2, 9, 35]. In macroalgae, the expression of the HSP genes has been investigated in several genus as they usually live in intertidal zone which characterized by regular and extreme changes in abiotic conditions, based on tidal influence [14]. However, the studies of HSP90 in algae are relatively limited [21, 27].* Chlorella vulgaris*, as an important economical species, is also an important model species in studies on stress responses [30]. Therefore, the role of* Chlorella vulgaris* HSP90 (designated CvHSP90) in response to adverse environment was investigated in this work.

In photosynthetic eukaryotes, Hsp90 family proteins are divided into five types with localization in different cellular compartments including nucleoplasm, chloroplasts, mitochondria, ER, and cytoplasm [6]. Sequence analysis of the deduced AA sequence showed that CvHSP90 contains the five typical signature motifs of the HSPs [10, 11, 36]. Moreover, the MEEVD motif which is cytoplasmic Hsp90-specific is identified at the C-terminus. The MEEVD motif participates in the formation of a Hsp90/Hop/Hsp70 protein complex concerning the assembly of steroid hormone receptors by associating with cytoplasmic Hsp70 through a Hsp-organizing protein (Hop). In addition, multiple alignment and phylogenetic analysis showed high similarities among the deduced amino acid sequence of CvHSP90 and other known HSP90s (more than 75% similarity in all the matches), especially with those from Mamiellophyceae* Micromonas *sp.* RCC299* and* Micromonas pusilla CCMP1545* (85% similarity in each match). In terms of sequence alignment, structure comparison, and phylogenetic analysis, CvHSP90 was confirmed to be a cytoplasmic Hsp90.

It is a main character for almost all organisms studied that the maximum expression of HSPs is 10–15°C above optimum growth temperature [18]. Moreover, it has been proved that the temperature treatment in the range 10–15°C below optimum growth temperature may also lead to the maximum expression of HSP90 [27]. Given that the normal growing temperature range of* C. vulgaris* is 20–25°C, in which the expression level of HSP90s is always low, it can be expected that the maximum CvHSP90 expression would be in the ranges of 35–40°C and 5–10°C, which was consistent with our results. In macroalgae, short time treatment in temperature higher than normal growth temperature can induce the expression of HSPs, which is upregulated by increased thermal stresses and then decreased progressively after the expression profile reached the maximum [37]. In this work, we observed that 45°C treatment which is 15°C over the optimum temperature led to the decrease of CvHSP90 expression, which may be caused by immediate deactivation or strong inhibition of enzyme-related mRNA synthesis and expression in* C. vulgaris* [19]. Furthermore, the time-dependent pattern of CvHSP90 expression in a heat shock was observed. In heat shock at 35°C, the expression increased progressively in 1 h and reached the maximum in 7 h and then declined gradually. Variance analysis indicated that the CvHSP90 gene expression in 6 or 7 hours after challenge was significantly higher (*P* < 0.01) than those in other time points. These results suggested that the CvHSP90 expression is related to the response to thermal stress and heat shock time and may play an important role in the mechanism against the adverse environmental stresses. In natural environment,* C. vulgaris* usually grows in salinity 20–30‰. Its utilization in wastewater treatment suggests that it is highly resistant to a range of salinity. In this work, the result showed that the mRNA expression levels of CvHSP90 varied with salinity and suggested its function in resistance to salinity stress. In addition, the CvHSP90 expression at different times under a fixed level of salinity stress needs to be further studied.

## 5. Conclusions

The CvHSP90 gene can be expressed in response to challenges in thermal stress, heat shock time, and salinity. In a stressful environment, changes in HSP90s expression level are more sensitive than those of growth rate, death rate, and reproductive rate for monitoring environmental stresses. Therefore, CvHSP90 can be used as a potential biomarker in practice to monitor environment changes.

## Figures and Tables

**Table 1:** PCR primers used in this study.

Primers	Sequence (5′-3′)	Sequence information
P1	GNGTGTTCATCATGGACAAYTGYGA	Homology cloning primer
P2	TTCATGATGCKYTCCATGTTNGC	Homology cloning primer
P3	GCCCCTCAACATCTCCCGCG	3′-RACE primer
P4	ACCTGCCCCTCAACATCTCCC	3′-RACE primer
adaptor	GGCACGCGTCGACTAGTAC	3′-RACE primer
P5	TTTTGTCCTCCTCGGTCTCG	5′-RACE primer
P6	GCCCTCCTTGGTCACGCTCA	5′-RACE primer
Oligo(dG)-adaptor	GGCACGCGTCGACTAGTACG10	5′-RACE primer
P7	GGCCAGTTCGGCGTGGGTTT	Real time gene primer
P8	AAGCGACGGGTTGACGGTGT	Real time gene primer
P9	AACTACGAGCTGCCAGACGG	Real time actin primer
P10	GAAGACAAGACGATGCTGAGGG	Real time actin primer
M13-47	CGCCAGGGTTTTCCAGTCACGAC	Sequencing primer
RV-M	GAGCGGATAACAATTTCACACAGG	Sequencing primer

**Table 2:** Sequences used for multiple alignment and phylogenetic analysis.

Species	Taxonomy	Accession	Similarity%
*Arabidopsis thaliana *	Streptophyta; Magnoliophyta; Eudicotyledons	AAN31859	77%
*Chlamydomonas reinhardtii *	Chlorophyta; Chlorophyceae	XP_001695264	79%
*Chlorella vulgaris *	Chlorophyta; Trebouxiophyceae	JQ655149	100%
*Glycine max *	Streptophyta; Magnoliophyta; Eudicotyledons	ADC45395	77%
*Haematococcus pluvialis *	Chlorophyta; Chlorophyceae	JN627245	79%
*Hordeum vulgare *subsp.* vulgare *	Streptophyta; Magnoliophyta; Liliopsida	BAJ86355	78%
*Micromonas pusilla CCMP1545 *	Chlorophyta; Mamiellophyceae	XP_003058104	85%
*Micromonas *sp. *RCC299*	Chlorophyta; Mamiellophyceae	XP_002499727	85%
*Nicotiana benthamiana *	Streptophyta; Magnoliophyta; Eudicotyledons	AAR12194	77%
*Pennisetum glaucum *	Streptophyta; Magnoliophyta; Liliopsida	ADP89125	78%
*Physcomitrella patens *subsp.* patens *	Streptophyta; Bryophyta;	XP_001777414	77%
*Sorghum bicolor *	Streptophyta; Magnoliophyta; Liliopsida	XP_002444804	77%
*Thellungiella halophila *	Streptophyta; Magnoliophyta; Eudicotyledons	BAJ33984	78%
*Triticum aestivum *	Streptophyta; Magnoliophyta; Liliopsida	ADF31758	78%
*Triticum urartu *	Streptophyta; Magnoliophyta; Liliopsida	ADF31773	78%
*Vitis pseudoreticulata *	Streptophyta; Magnoliophyta; Eudicotyledons	ABW96308	77%
*Vitis vinifera *	Streptophyta; Magnoliophyta; Eudicotyledons	CAN62488	78%
*Volvox carteri* f. *nagariensis *	Chlorophyta; Chlorophyceae	XP_002947115	79%
*Zea mays *	Streptophyta; Magnoliophyta; Liliopsida	NP_001170475	76%
